# Reward Processing as an Indicator of Vulnerability or Compensatory Resilience in Psychoses? Results From a Twin Study

**DOI:** 10.1016/j.bpsgos.2022.01.002

**Published:** 2022-01-21

**Authors:** Mette Ødegaard Nielsen, Egill Rostrup, Rikke Hilker, Christian Legind, Simon Anhøj, Trevor William Robbins, Barbara J. Sahakian, Birgitte Fagerlund, Birte Glenthøj

**Affiliations:** a Center for Neuropsychiatric Schizophrenia Research and Centre for Clinical Intervention and Neuropsychiatric Schizophrenia Research, Mental Health Centre Glostrup, Copenhagen, Denmark; b Department of Clinical Medicine, Faculty of Health and Medical Science, University of Copenhagen, Copenhagen, Denmark; c Department of Psychology, University of Copenhagen, Copenhagen, Denmark; d Functional Imaging Unit, Department of Clinical Physiology, Nuclear Medicine and PET, Rigshospitalet, Glostrup, Denmark; e Department of Psychology, Behavioural and Clinical Neuroscience Institute, University of Cambridge, Cambridge, United Kingdom; f Department of Psychiatry, Behavioural and Clinical Neuroscience Institute, University of Cambridge, Cambridge, United Kingdom

**Keywords:** Functional magnetic resonance imaging, Prediction error, Reward, Schizophrenia, Twins, Vulnerability indicator

## Abstract

**Background:**

Findings of reward disturbances in unaffected relatives of patients with schizophrenia suggest reward disturbances as an endophenotype for schizophrenia. Twin studies, where 1 twin has been diagnosed with a schizophrenia spectrum disorder, can further explore this.

**Methods:**

We used Danish registries to identify twin pairs with at least 1 twin having a schizophrenia spectrum disorder diagnosis and control twin pairs matched on age, sex, and zygosity. The analyses included data from 34 unaffected co-twins (16 females), 42 probands with schizophrenia spectrum disorder (17 females), and 83 control twins (42 females). Participants performed a modified incentive delay task during functional magnetic resonance imaging. Whole-brain group differences were analyzed by performing comparisons between co-twins and control twins. Correlations with cognitive flexibility were tested.

**Results:**

Compared with control twins, co-twins showed no differences in striatal regions, but increased signal in the dorsolateral prefrontal cortex (DLPFC) during missed target contrast was observed. In co-twins, increased DLPFC signal was associated with lower intra-extra dimensional set-shifting scores indicative of higher cognitive flexibility.

**Conclusions:**

Unaffected co-twins did not have decreased striatal activity during anticipation as previously reported for patients with schizophrenia. Instead, they showed increased activity in the DLPFC during evaluation of missed target contrast, which correlated with their level of cognitive flexibility. Unaffected co-twins had no diagnosis at a mean age of 40 years. This could indicate that greater cognitive flexibility and increased activity in the right DLPFC during processing of unexpected negative outcome represents a compensatory resilience mechanism in predisposed twins.

SEE COMMENTARY ON PAGE 8

A consistent finding in antipsychotic-naïve (1) and medicated patients with schizophrenia is alterations in the brain reward system [reviewed in (2)]. In studies using functional magnetic resonance imaging (fMRI), areas normally activated in the ventral striatum during anticipation of rewarding and punishing events show reduced contrast signal in patients with schizophrenia. Likewise, an altered response during outcome evaluation and goal-directed behavior has been observed in several regions, where the contrast signal in the medial prefrontal cortex, dorsolateral prefrontal cortex (DLPFC), or striatum is either decreased (3, 4, 5, 6) or increased (7,8).

Alterations in the reward system have been suggested as an endophenotype, or a vulnerability indicator, for schizophrenia because alterations have been found in prodromal patients (9,10), first-degree relatives (11), unaffected siblings (12), and the offspring of patients with schizophrenia (13). Grimm *et al.* (12) found that unaffected relatives (siblings, parents, and offspring) of patients with schizophrenia showed reduced striatal activity during reward anticipation compared with healthy control subjects. These findings were replicated in a cohort of unaffected siblings, and although no significant psychopathological symptoms were present, reduced activation during reward anticipation correlated with subclinical negative symptoms. In addition, siblings showed increased activation in the ventral striatum and orbitofrontal cortex compared with control subjects during reward outcome evaluation. Most recently, a study found that striatal activation during reward anticipation decreased with age in the offspring of patients with schizophrenia, which was not the case in healthy control subjects (13).

To date, most studies comparing healthy relatives to patients with schizophrenia have examined vulnerability indicators by focusing on alterations in the reward system similar to the ones found in patients and linked these alterations to subtle levels of psychopathological symptoms (11, 12, 13). A group of unaffected adult relatives, however, can be viewed in another way: despite having a genetic predisposition, they did not develop a severe psychiatric illness and may thus possess resilience factors or compensatory mechanisms. Interactions between cognitive deficits and dysfunctions in reward processing have been suggested as synergistic factors for developing schizophrenia (14). Thus, good cognitive abilities affecting reward processing may as well serve as modulators and protect against development of severe psychiatric symptoms. Flexibility in thinking is important for correctly updating beliefs during mismatch between predicted and expected outcome, and cognitive functions are suggested to play a role as top-down regulators of more basic reward processing (15). Specifically, better cognitive flexibility has been associated with increased basal ganglia connectivity of the default mode network (16), whereas less flexibility in thinking is found in patients with delusions (17).

In this study, we compared brain activity during reward processing in unaffected co-twins and healthy control twins to test the following hypotheses: 1) unaffected co-twins will have decreased signal in striatal regions in the anticipation contrasts of the reward task, which will constitute a stable vulnerability indicator; 2) unaffected co-twins also will show other deviations in the reward system, which could be considered to reflect compensatory resilience mechanisms; and 3) in unaffected co-twins, vulnerability indicators will correlate with subtle psychopathology, whereas a possible resilience mechanism will correlate with cognitive abilities.

## Methods and Materials

The data are from a multimodal twin study approved by the Danish National Committee on Biomedical Research Ethics (File No. H-2-2010-1289), which was conducted in accordance with the Declaration of Helsinki II. All participants provided written informed consent. Data on other measures from the twin cohort have previously been published (18, 19, 20, 21).

### Participants

By coupling information from the Danish Twin Registry to the Danish Psychiatric Central Research Register, monozygotic and dizygotic twins with a schizophrenia spectrum disorder diagnosis (probands) were identified and invited to participate in the study along with their co-twin. A sample of twins matched on age, sex, and zygosity but without any registered psychiatric diagnosis was invited as well. All participants went through a diagnostic interview using the Schedule of Clinical Assessment in Neuropsychiatry version 2.1. Co-twins and control twins who met ICD-10 diagnostic criteria or received psychiatric medication were excluded from the analyses. All participants were rated using the Positive and Negative Syndrome Scale (PANSS) (22), while level of functioning was estimated using the Global Assessment of Functioning (GAF) scale (23).

### Experimental Design and Tasks

To elicit striatal activation in response to cues indicating monetary gain and loss, we used a modified variant of the monetary incentive delay task described by Knutson (24) and modified by Cooper and Knutson (25). Participants were initially presented with a cue indicating one of six trial conditions representing either uncertain gain or loss, certain gain or loss, or two neutral conditions. In neutral conditions, the outcome was no monetary gain or loss. In certain conditions, participants were sure of either gaining or losing 7 euros, whereas in uncertain conditions, winning or losing 7 euros depended on their ability to press the button while a target was visible on screen. Thus, in uncertain gain, the outcome could be either gaining 7 euros (hit) or not gaining (miss), and in uncertain loss, the outcome could be no loss (hit) or loss of 7 euros (miss). After cue representation (2.5 s) and a short delay (2–4 s), a visual target briefly appeared on the screen, and participants were instructed to press a button while the target was visible. Initial target time was 300 ms but was individually adjusted to reach an overall hit rate of approximately 66%. After another delay (2–4 s), feedback on gain or loss in the specific task as well as the total monetary outcome was shown (5 s). The session lasted 18 minutes and comprised 72 trials, each of which lasted 15 s. Participants practiced the task for 10 minutes before going to the scanner. To keep motivation high, participants were given the amount of money they won immediately after the scan was over.

Premorbid intelligence was estimated using the Danish Adult Reading Test (26), and cognitive flexibility was measured with the intra-extra dimensional (IED) set shift from the Cambridge Neuropsychological Test Automated Battery using the total error-adjusted measure and the IED extradimensional shift error score (26,27). This test estimates cognitive/attentional flexibility and the ability to switch from a simple rule (intradimensional) to a more complex/abstract rule (extradimensional). Fewer errors indicate better performance in the test and higher cognitive flexibility.

### Imaging

Functional and structural MRIs were performed with a Philips Achieva 3.0T whole-body MRI scanner (Philips Healthcare) using an eight-channel SENSE head coil (InVivo). At each scanning session, whole-brain three-dimensional high-resolution T1-weighted structural images were acquired for anatomical reference (repetition time = 10 s, echo time = 4.6 ms, flip angle = 8°, voxel size = 0.79 × 0.79 × 0.80 mm). For each participant, we acquired 540 whole-brain functional echo-planar images per run (38 slices, thickness = 2.4 mm, voxel size = 2.4 × 2.9 × 2.9 mm, flip angle = 75°, repetition time = 2 s, echo time = 25 ms).

### Data Analysis

fMRI analyses were conducted using tools from the FMRIB Software Library (John Radcliffe Hospital). Images were corrected for slice-timing effects, while spatial smoothing was performed with a 5-mm full width at half maximum Gaussian kernel and low-frequency noise was reduced using a high-pass filter of 200 s. The functional images were coregistered with the three-dimensional anatomical images, and both were transformed into Montreal Neurological Institute space.

For the first-level analyses, the reward paradigm was modeled with 15 predictors, 6 of which were the cues indicating the tasks’ conditions: certain and uncertain win, certain and uncertain loss, and two neutral ones. There were an additional 7 predictors for the different outcomes: certain gain, certain loss, uncertain gain (winning in uncertain gain trial), uncertain loss (losing in uncertain loss trial), zero hit (not losing in uncertain loss trials), zero miss (not winning in uncertain gain trial), and zero neutral (expected outcome of zero in neutral trials). Finally, there were two predictors of no interest indicating the button press for behaviorally important (uncertain) and unimportant (certain and neutral) events. All explanatory variables were convolved with the hemodynamic response function, and their temporal derivative was added.

Individual contrast images were computed. Anticipation contrasts were anticipation of winning (cues indicating certain and uncertain win vs. neutral cues), anticipation of losing (cues indicating certain and uncertain loss vs. neutral cues), and anticipation of unpredictable outcome (cues indicating uncertain win and loss vs. neutral cues). For outcome evaluation, four contrasts were defined: monetary gain (certain and uncertain gain vs. zero neutral), monetary loss (certain and uncertain loss vs. zero neutral), hit target (uncertain gain and zero hit vs. zero neutral), and missed target (uncertain loss and zero miss vs. zero neutral). Six motion regressors were also included to correct for three-dimensional motion effect.

Our main outcome was whole-brain voxelwise comparison of all 7 contrasts between unaffected co-twins and control twins. In addition, whole-brain comparisons for all contrasts were performed between probands and control twins and between probands and co-twins.

The images were thresholded using clusters determined by *Z* > 3.1 and by a corrected cluster significance threshold of *p* = .05 (28), corresponding to uncorrected *p* < .001 and clusters >15 voxels.

### Secondary Region of Interest Analyses

Using the same paradigm, we have previously found a decreased signal in striatal regions in the contrast anticipation of unpredictable outcome in patients with schizophrenia (1). For this contrast, values for the mean percent signal change were therefore extracted from the bilateral nucleus accumbens, the head of the caudate nucleus, and a central part of the putamen. In accordance with previously published studies (29, 30, 31, 32), the radius for each region of interest (ROI) sphere was defined as 6 mm centered in the Montreal Neurological Institute coordinates ±10, 14, −6; ±10, 12, 8; and ±22, 4, 4. Comparison between all three groups was performed and in case of significant group differences, the extracted values would be correlated with psychopathology (PANSS scores). Likewise, the mean percent signal change would be extracted for correlation with the cognitive measures (Danish Adult Reading Test and IED set-shifting) from any prefrontal regions showing group difference between unaffected co-twins and control twins.

### Additional Analyses on the Schizophrenia-Only Sample

Because of the diversity in the schizophrenia spectrum diagnosis, analyses on group differences were repeated including only the 25 probands with schizophrenia or schizoaffective disorder and the 20 co-twins whose proband twins had these diagnoses.

### Statistics

Demographic and psychopathological data for the three groups was compared using the χ^2^ test (sex and zygosity), one-way analysis of variance (ANOVA)/*t* test (age), and Mann-Whitney *U* test (PANSS and GAF). For the behavioral data, total monetary gain was compared with one-way ANOVA. Hit rate and reaction time were analyzed using 3 × 6 ANOVA with group as the between-subject factor and trial type as the within-subject factor. Student’s *t* test was used for post hoc analyses. Correlations were performed using Spearman’s correlation coefficient, and significance level was corrected for multiple comparisons (*p* < .017).

## Results

**Figure 1:**
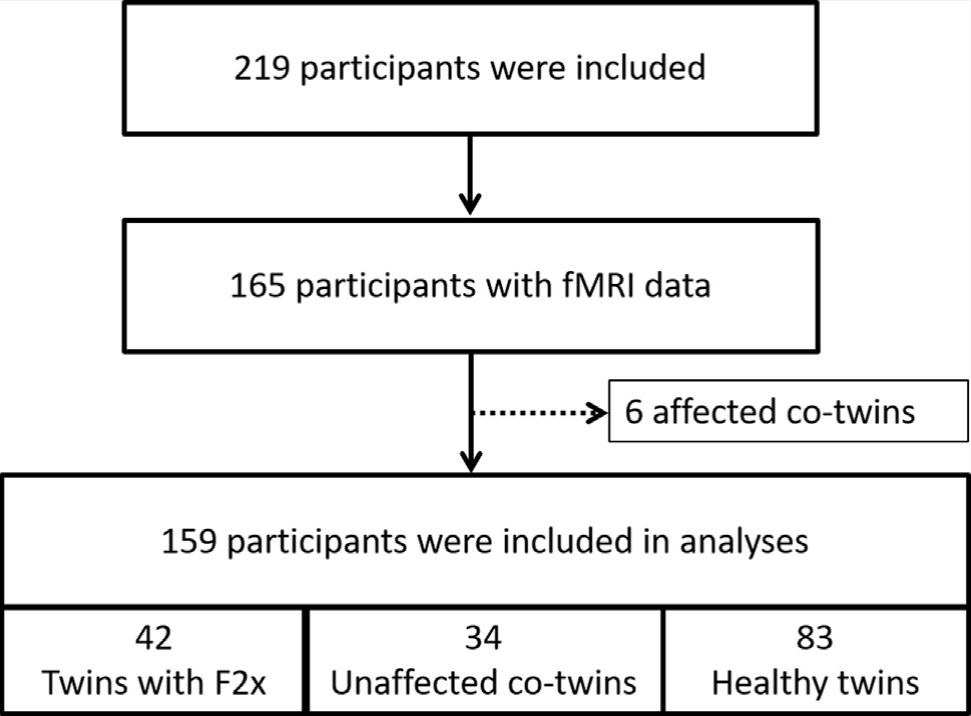
Flowchart of study participants. F2x, schizophrenia spectrum diagnoses; fMRI, functional magnetic resonance imaging.

We included 219 individuals, but fMRI scans were only available for 165 of them. Furthermore, 6 co-twins met the criteria for a psychiatric diagnosis and were excluded because they were not unaffected. Thus, fMRI data of sufficient quality were available for 159 participants: 34 co-twins, 42 probands, and 83 control twins (Figure 1). They were between 18 and 60 years old and there were no group differences in age, sex, or zygosity. The probands were diagnosed with schizophrenia (*n* = 24), schizotypal disorder (*n* = 9), brief psychotic disorders (*n* = 8), schizoaffective disorder (*n* = 1), and unspecified psychosis (*n* = 1). Seventeen probands received second-generation antipsychotic medication, 3 received first-generation antipsychotic medication, 2 received a combination of first- and second-generation antipsychotic medication, and 20 did not receive any antipsychotic medication at the time of examination. One-way ANOVA showed a group difference in all PANSS scores, and post hoc comparison showed that co-twins had a subtle, but significantly higher, level of psychopathology than the healthy control twins for PANSS total, PANSS positive, and PANSS general (all *p* values < .05). Likewise, they had a lower level of function as measured on GAF (*p* = .001). There were no differences between co-twins and control twins in cognitive measures (Table 1).

**Table 1:** Demographics

Characteristic	Probands, *n* = 42	Co-twins, *n* = 34	Control Twins, *n* = 83	Co-twins><Control Twins, *p* Value
Age, Years, Mean (SD)	39.5 (9.9)	39.5 (11.3)	41.3 (10.3)	.804
Sex, Female/Male, *n*	17/25	16/18	42/41	.728
Monozygotic/Dizygotic, *n*	23/19	17/17	34/49	.371
PANSS Total, Mean (SD)	59.6 (18.9)	35.9 (7.1)	31.6 (2.1)	.005^a^
Positive	14.1 (6.0)	8.5 (2.5)	7.2 (0.6)	.024^a^
Negative	15.5 (7.1)	8.6 (2.5)	7.6 (1.2)	.074
General	30.0 (8.8)	18.8 (4.0)	16.8 (1.4)	.023^a^
GAF, Mean (SD)	55 (14.9)	78 (11.1)	86 (7.1)	.004^a^
Antipsychotic Medication, Atypical/Typical/Both, *n*	17/3/2	0/0/0	0/0/0	–
DART, Mean (SD)	24.7 (9.5)	24.5 (7.2)	23.5 (6.7)	.900
IED Total Error Adjusted, Mean (SD)	34.1 (22.7)	27.8 (20.4)	23.4 (19.8)	.630
IED EDS Stage, Mean (SD)	17.4 (11.4)	15.5 (11.4)	12.3 (11.0)	.420
Failed EDS Stage, %	45%	32%	24%	.358
Amount of Money Won, DKR, Mean	421	476	466	.615

Data on demographic, psychopathology, selected cognitive scores, and monetary gain during the reward task. All three groups are shown, and *p* values are presented for comparisons between co-twins and control twins. Significant differences were also found between probands and control twins in psychopathology, level of function, and IED total errors adjusted (*p* < .05).

DART, Danish Adult Reading Test; DKR, Danish kroner; EDS, extradimensional shift; GAF, Global Assessment of Functioning; IED, intra-extra dimensional; PANSS, Positive and Negative Syndrome Scale.

a Significant difference between co-twins and control twins (*p* < .05).

### Behavioral Data

There were no group differences in the amount of money won during the game (Table 1). For the hit rate, there was a main effect of trial type but no main effect of group and no group × trial-type interaction. We found a higher hit rate in the uncertain gain and uncertain loss trials than neutral and certain trials.

For reaction time, there was an effect of group and trial type and a group × trial-type interaction. Probands were slower than co-twins and control twins, and for all three groups, there was a slower reaction time in the neutral trials. In addition, control twins had a slower reaction time in the certain gain and loss trials, which was not the case for co-twins and probands.

For IED set-shifting, proband twins had the highest number of total errors adjusted, which was significant compared with control twins. Although co-twins had more errors than control twins, this difference was not significant (Table 1). For the IED scores, there was a tendency for a bimodal distribution in all three groups due to the pass/fail nature of the IED test and the estimation of errors for stages not completed, which is in line with previous findings (33).

### Co-twins Compared With Control Twins

During the anticipation phase, we found no differences between co-twins and control twins using a whole-brain voxelwise comparison.

**Figure 2:**
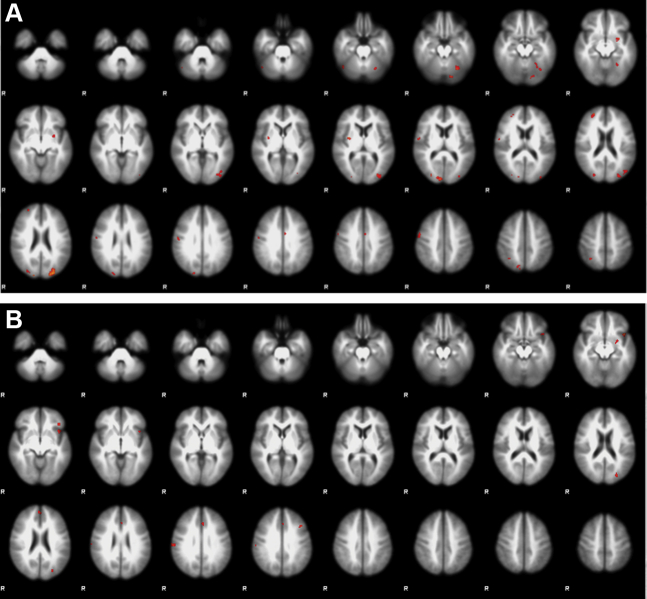
Whole-brain group comparison. Voxelwise comparison during the missed target contrast **(A)** between unaffected co-twins and control twins and **(B)** between unaffected co-twins and probands. The images were thresholded using clusters determined by *Z* > 3.1 and a corrected cluster significance threshold of *p* = .05. R, right.

During outcome evaluation, we found an increased signal in unaffected co-twins compared with control twins in the missed target contrast, which included an area in the right DLPFC, and smaller clusters in the cerebellum, putamen, and occipital and parietal cortices (Figure 2A and Table S1). In the monetary loss contrast, unaffected co-twins showed an increased signal compared with control twins in a small region in the left cerebellum (not shown). There were no group differences in the monetary gain or hit target contrasts.

### Probands Compared With Co-twins/Control Twins

During the anticipation phase, we found no differences between probands and control twins or between probands and co-twins using a voxelwise approach.

During outcome evaluation, we found no differences between probands and control twins in any contrast. Compared with probands, co-twins showed increased signal during the missed target contrast in several small clusters in the cerebellum, putamen, and occipital, parietal, and medial frontal cortices (Figure 2B and Table S2). There were no differences in the other outcome evaluation contrasts.

### ROI Analyses

**Figure 3:**
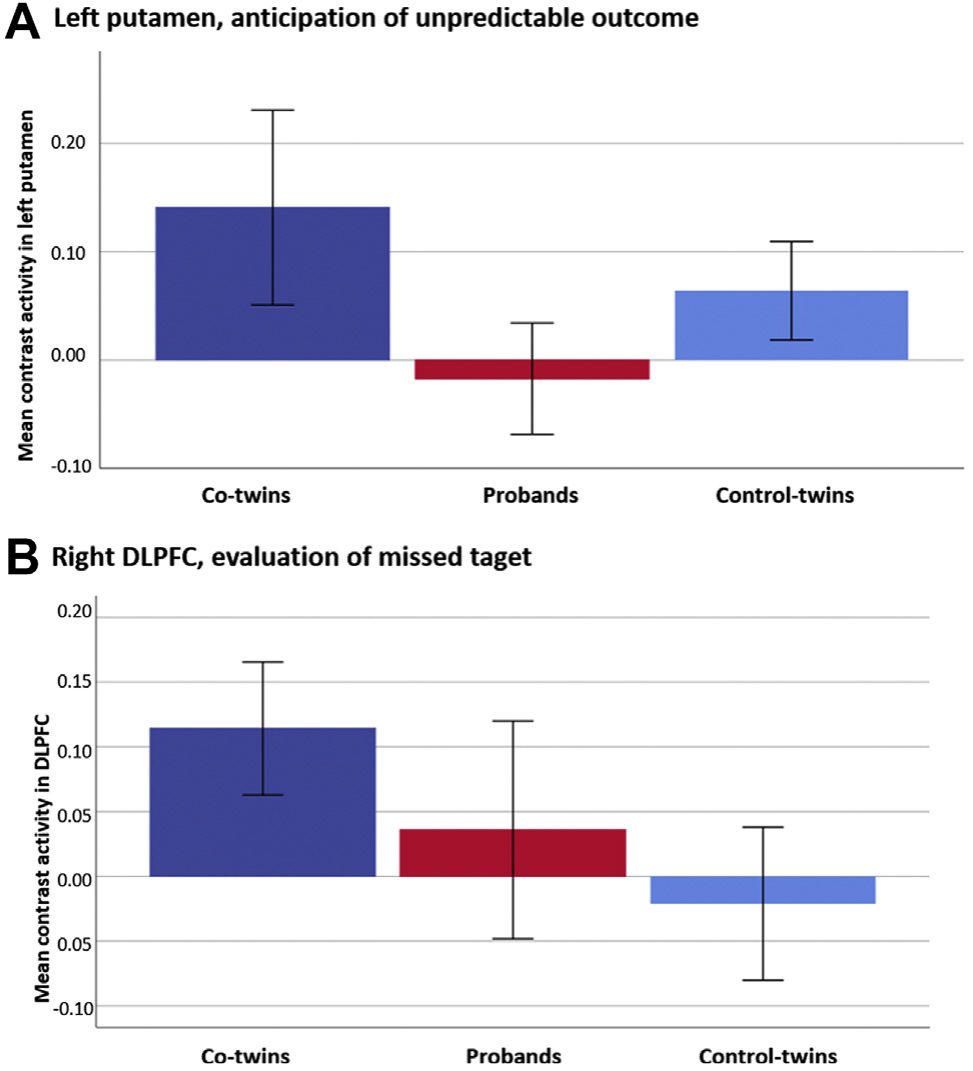
Regions of interest. Mean contrast activity extracted from **(A)** the left putamen showing significant group difference between probands (red) and co-twins (dark blue) and control twins (light blue) in anticipation contrast of unpredictable outcome and from **(B)** the dorsolateral prefrontal cortex (DLPFC) showing significant group difference between co-twins (dark blue) and control twins (light blue) during evaluation of the missed target contrast. Error bars show 95% confidence interval.

The contrast signal indicating anticipation of unpredictable outcome extracted from striatal ROI showed a group difference in the left putamen only (*F*_2,156_ = 4.43, *p* = .013). Although this result did not survive correction for multiple comparison, post hoc analyses were performed and showed that the group difference was caused by decreased activity in probands compared with co-twins (*t*_74_ = 2.94, *p* = .004) and control twins (*t*_123_ = 2.01, *p* = .047) (Figure 3A). In probands, the contrast signal in the left putamen showed a negative correlation with PANSS total (ρ = −0.4, *p* = .007), PANSS positive (ρ = −0.4, *p* = .007), PANSS negative (ρ = −0.36, *p* = .016), and PANSS general (ρ = −0.31, *p* = .04) scores.

**Figure 4:**
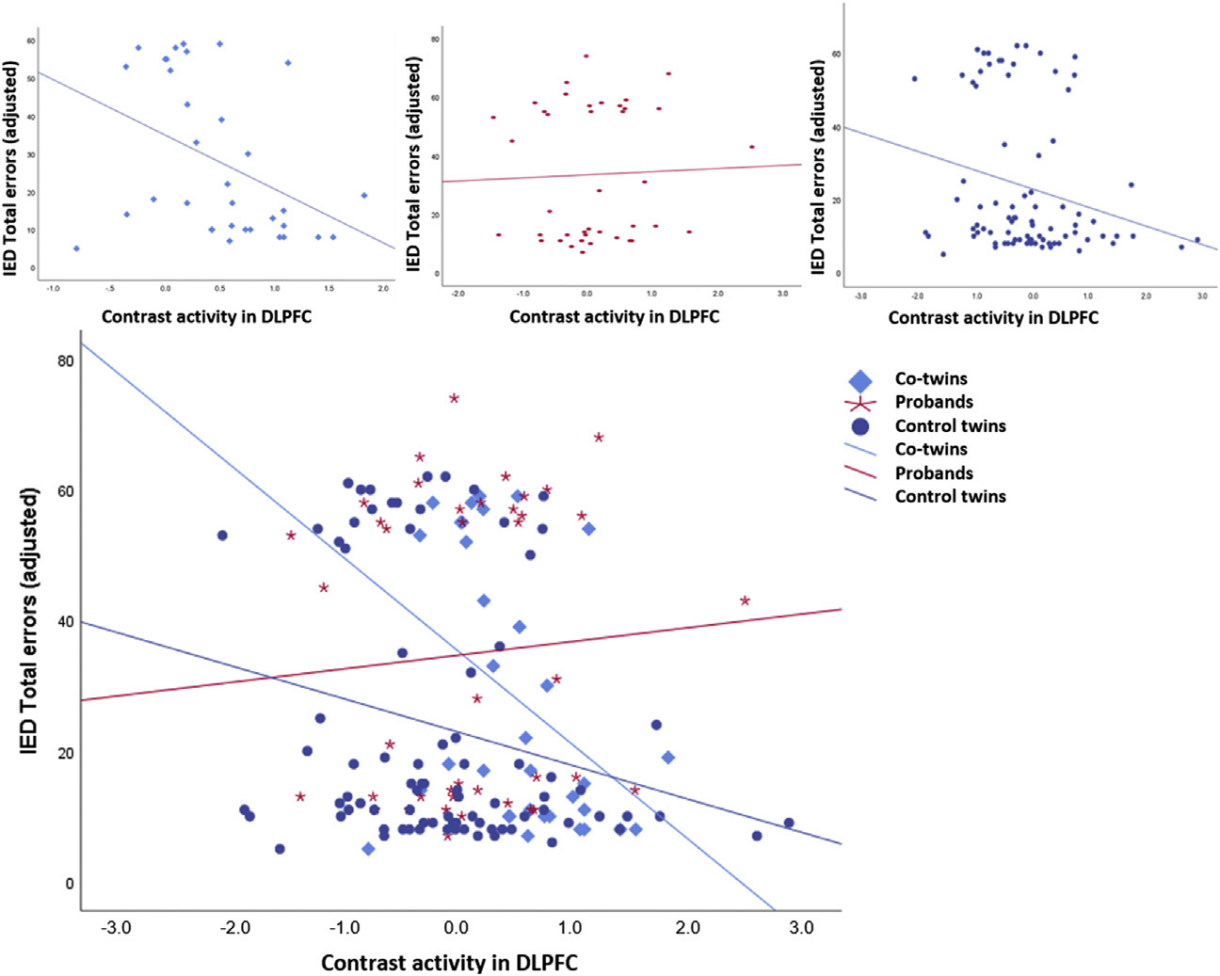
Correlations. Illustration of the correlation between contrast activity from the dorsolateral prefrontal cortex (DLPFC) and intra-extra dimensional (IED) total errors adjusted for each of the groups: Unaffected co-twins (ρ = −0.45, *p* = .008), probands (ρ = 0.15, *p* = .34), and control twins (ρ = −0.255, *p* = .02).

For the DLPFC cluster showing a significant group difference, the missed target contrast signal was extracted for all participants (Figure 3B). Higher missed target contrast signal in the DLPFC was correlated with lower IED total errors-adjusted score and the IED extradimensional shift errors score in unaffected co-twins (ρ = −0.45, *p* = .008; ρ = −0.39, *p* = .022) and control twins (ρ = −0.26, *p* = .020; ρ = −0.21, *p* = .049). Only the correlation with IED total errors adjusted in co-twins survived correction for multiple comparison (*p* < .017) (Figure 4). There were no correlations in the probands and no correlations with the Danish Adult Reading Test in any of the groups. The values were also separated by zygosity (see Figure S1).

### Schizophrenia-Only Sample

Whole-brain comparison showed increased signal in the occipital cortex in unaffected co-twins compared with control twins in the missed target contrast (Figure S2 and Table S3). There were no other differences between co-twins and control twins. Compared with control twins, probands showed decreased signal during anticipation of winning in the right prefrontal cortex and increased signal in the parietal cortex during evaluation of monetary loss (Figures S3 and S4).

For the ROI analyses, one-way ANOVA showed a group difference in the left putamen (*F*_2,125_ = 4.8, *p* = .010) and in the nucleus accumbens bilaterally (right: *F*_2,125_ = 3.9, *p* = .023; left: *F*_2,125_ = 4.2, *p* = .017). In all three ROIs, this was caused by decreased activity in probands compared with control twins (all *p* values < .02) and in probands compared with co-twins in the left putamen and accumbens (*p* < .04) (Figure S5). In none of the striatal ROIs did we find a decreased activity in co-twins compared with control twins (all *p* values > .1 and effect sizes < 0.26).

In the DLPFC, there was a group difference (*F*_2,125_ = 3.3, *p* = .038) caused by increased signal in co-twins compared with control twins (*p* = .013, effect size = 0.63).

## Discussion

Contrary to our expectations, unaffected co-twins did not show decreased reward activity in striatal regions during any anticipation contrasts. Instead, compared with control twins and probands, co-twins showed increased activity in several brain areas during evaluation of trials with unexpected negative outcome. In addition, increased activity in the DLPFC during these trials was associated with a better performance in IED set-shifting for co-twins and control twins, but not for probands.

In our whole-brain analyses focusing on outcome evaluation, we found an altered response in unaffected co-twins only during the missed target contrast. This is interesting because humans constantly use recent experiences to update beliefs about the world. Fletcher and Frith (34) suggested that formation of delusional ideas occurs when the newly accumulated evidence about the world does not appropriately update these beliefs. The term negative prediction error is used for situations where there is a discrepancy between the expected and the actual outcome, and the underlying neural coding involves striatal and several prefrontal cortical areas. In some studies, patients with schizophrenia showed altered brain activity during prediction errors in relevant brain regions such as the striatum and areas in the prefrontal cortex, and in line with this theoretical framework, these abnormalities have been associated with delusions (7,35,36). There may be mechanisms, however, that work against developing psychotic symptoms. In prodromal patients with less pronounced symptoms, Ermakova *et al.* (37) found disrupted brain activity in the midbrain but intact frontal prediction error signaling and suggested that this may prevent mild symptoms from becoming severe. In line with this, Corlett and Fletcher (38) found an association between nonclinical schizotypal experiences and aberrant striatal prediction error signal in healthy individuals, but these experiences were not distressing in individuals who showed high activity in the DLPFC during prediction error. Although our missed target contrast may not exactly be the same as prediction error, it can be categorized as unpredicted negative outcome because of the high hit rate and may therefore involve similar networks as prediction error. Increased activity in the DLPFC was also present in the ROI analyses on the most genetically predisposed twins, i.e., unaffected co-twins whose siblings had developed schizophrenia or schizoaffective psychoses. We believe this supports the idea that increased DLPFC activity may be a possible protective/compensatory resilience factor for developing psychoses.

A previous study on healthy volunteers demonstrated a correlation between IED performance and connectivity between the DLPFC and ventral striatum (39). In our study, unaffected co-twins showed increased activity in the right DLPFC during the missed target contrast, which in co-twins and control twins was most pronounced in those with better cognitive flexibility. Furthermore, unaffected co-twins showed increased activity in the missed target contrast in several other brain regions, especially in the cerebellum. The cerebellum is involved in monitoring performance and adapt behavior, and increased cerebellar activity may be associated with increased emotional and cognitive associative learning (40, 41, 42). We suggest that increased activity in multiple brain areas during processing of unexpected negative outcome and greater cognitive flexibility may represent a compensatory resilience mechanism in unaffected co-twins. Despite having a genetic predisposition for developing psychotic disorders, their vulnerability may be compensated by more flexible thinking, which may help them continuously to adjust and update their beliefs about the surrounding world and therefore not develop psychotic symptoms. In turn, increased activation of the DLPFC may enable increased cognitive flexibility. Longitudinal studies on high-risk individuals are necessary to examine the degree to which these measures are actually compensatory resilience factors. In the same group of clinical high-risk individuals, it may likewise be relevant to test whether training of cognitive flexibility works as a protecting factor against developing a psychotic episode and to what degree this is directly associated with DLPFC activity.

One of our primary aims was to examine whether alterations in the reward system can be understood as a vulnerability indicator of psychosis. For this reason, we expected that highly genetically predisposed co-twins would have decreased activity in striatal regions, as we, and others, have demonstrated in patients with schizophrenia (1,2). However, the group of unaffected co-twins did not show decreased reward signaling during any of the anticipation contrasts. This was the case even though their vulnerability was measurable in terms of slightly increased PANSS scores and decreased GAF, which was not found in previous studies on unaffected relatives (11, 12, 13). There are, however, other characteristics of the cohorts examined that may explain why these findings are different than the literature.

One important factor is the older age of our cohort. In previous studies, the mean age of the predisposed individuals was 14 to 33 years, whereas our co-twins had no psychiatric diagnoses at a mean age around 40 (range, 18–60) years. This may be important in two ways. First, there is some evidence of a clear effect of age on reward-related brain activity, especially in the striatum (43). Furthermore, Vink *et al.* (44) demonstrated that the typical shift in striatal activation from reward receipt to reward anticipation in young adults disappears with healthy aging, which is consistent with the age-related decline in striatal dopamine availability (45). This may, to some degree, explain why decreased striatal activity may be more pronounced in cohorts of young patients and aging may therefore reduce this signal as a vulnerability indicator. Second, it may be the case that the most vulnerable co-twins have already developed symptoms to the extent that they had already received a psychiatric diagnosis and were therefore excluded. This was, in fact, the case for 6 of the co-twins (14%) in our scanned sample. Excluding these subjects left us with the most resilient individuals and, according to the hypotheses of altered reward activity as a vulnerability indicator, these remaining co-twins would have the most normal reward system.

Another reason why we found no alterations in striatal activity in our sample of co-twins may be that certain brain functions change over time. Thus, some of the co-twins may have had an altered reward response in the striatum earlier in their lives, perhaps during a more vulnerable period, but which has now normalized. Such a change over time has been shown for other brain regions in siblings of patients with schizophrenia. In a recent study, siblings of patients with schizophrenia showed changes similar to the affected sibling in early childhood, but during adolescence, these alterations normalized as their brains developed (46). There are, to our knowledge, no studies describing the long-term stability of reward responses in healthy individuals, patients with schizophrenia, or patients who are at ultra-high risk of psychoses. This generates new research questions to be addressed. How stable is the reward response in a healthy population in the long term? Is the alteration in striatal reward response in patients a state or a trait marker, or maybe even a predictive marker for later development of psychoses in ultra-high-risk patients? Or do some compensatory mechanisms in the network normalize these initial vulnerability indicators and therefore serve as resilience factors against developing a psychotic disorder?

In this sample of twins diagnosed with a schizophrenia spectrum diagnosis, we found decreased activity during anticipation of unpredictable outcome in only one of the striatal ROIs, the left putamen. We also found a correlation with symptomatology, which is in line with previous findings (1,47,48). There were, however, no group differences in other striatal ROIs. This may very well be related to diagnostic issues because analyses on the sample including only schizophrenia/schizoaffective probands showed decreased signal during anticipation of unpredictable outcome in the nucleus accumbens bilaterally. The analyses on the sample of co-twins whose proband twins had these diagnoses still showed no tendency of them having a decreased signal in striatal regions during any of the anticipation contrasts, not even in the ROI analyses.

In contrast to a previous study (12), we did not find group differences during evaluation of positive outcome. Using the same paradigm, we have not been able to demonstrate altered response in patients during outcome evaluation (1) and therefore did not use an ROI approach for outcome evaluation. Whole-brain analyses highlight the most significant group differences and are, as such, more explorative.

### Limitations

The sample was matched on age, sex, and zygosity, and the data were collected as a part of a large multimodal study. fMRI data were missing for a considerable number of participants, which made it impossible to keep a twin pair–to–twin pair match. Furthermore, it would have been interesting to investigate differences between monozygotic and dizygotic co-twins, but the numbers were too small to have the power for such analyses. We primarily used a whole-brain approach and investigated several contrasts, thereby increasing the risk of type I error. The unexpected findings in the DLPFC should therefore be considered as hypotheses generating and need replication in future studies.

### Conclusions

In this adult sample of unaffected co-twins highly genetically disposed to schizophrenia spectrum disorders, we did not find alterations in striatal regions during anticipation phases identical to previous findings in patients with schizophrenia, indicating that this is not a stable vulnerability indicator. Instead, we found increased activity in the DLPFC during unexpected negative outcome, which correlated with cognitive flexibility, pointing to possible compensatory resilience factors against developing severe psychiatric conditions, despite the genetic vulnerability.

## Supplementary Material

Supplementary Tables 1 and 2, and Supplementary Figures 1–5

Key Resources Tables
