# Supplementary material for: Reward Processing as an Indicator of Vulnerability or Compensatory Resilience in Psychoses? Results From a Twin Study
Source: Biol Psychiatry Glob Open Sci. 2022 Jan 21;3(1):47–55. doi: 10.1016/j.bpsgos.2022.01.002 (PMC9874133; doi:10.1016/j.bpsgos.2022.01.002)

# Reward Processing as an Indicator of Vulnerability or Compensatory Resilience? Results From a Twin Study

## *Supplement*

### Supplement table S1

Clusters with increased activity in whole brain comparison of the missed target contrast between unaffected co-twins and control twins, thresholded Z>3.1

| Area | | Voxels | Z-max | X | Y | Z |
|---|---|---|---|---|---|---|
| Left | Superior frontal gyrus (BA19) | 211 | 4.58 | -26 | -90 | 24 |
| | Cerebellum | 96 | 4.05 | -26 | -64 | -22 |
| | Putamen | 34 | 3.97 | -30 | 2 | -10 |
| | Occipital lobe | 32 | 4.19 | -14 | -82 | -18 |
| | Occipital cortex | 31 | 4.00 | -46 | -78 | 22 |
| Right | Visual associate cortex | 52 | 4.30 | 10 | -94 | 12 |
| | Frontal cortex (BA10) | 39 | 3.79 | 26 | 50 | 18 |
| | Putamen | 33 | 4.30 | 34 | -2 | 6 |
| | Precentral gyrus (BA6) | 28 | 3.67 | 56 | -2 | 44 |
| | Occipital cortex (BA19) | 25 | 3.56 | 26 | -88 | 20 |
| | Postcentral gyrus | 24 | 3.69 | 56 | -10 | 32 |
| | Visual associate cortex | 23 | 3.74 | 18 | -94 | 24 |
| | Primary motor cortex | 21 | 3.92 | 58 | -4 | 14 |
| | Cerebellum | 20 | 3.83 | 48 | -60 | -24 |
| | Visual associate cortex | 17 | 3.61 | 30 | -86 | 12 |
| | Parietal lobe | 17 | 3.59 | 14 | -78 | 46 |
| | Superior parietal lobe | 16 | 3.61 | 34 | -58 | 52 |
| | Cingulate gyrus (BA24) | 16 | 3.67 | -4 | 0 | 38 |

### Supplement table S2

Clusters with increased activity in whole brain comparison of the missed target contrast between unaffected co-twins and proband twins, thresholded Z>3.1

| Area | | Voxels | Z-max | X | Y | Z |
|---|---|---|---|---|---|---|
| Left | inferior front gyrus (BA47) | 62 | 4.26 | -44 | 16 | -10 |
| | Occipital lobe (BA19) | 35 | 4.07 | -26 | -80 | 22 |
| | Cingulate gyrus (BA32) | 27 | 3.88 | -2 | 26 | 30 |
| | Putamen | 23 | 3.71 | -24 | -2 | -10 |
| | Precentral gyrus (BA9) | 19 | 3.95 | -42 | 22 | 36 |
| Right | Inferior parietal lobe (BA40) | 43 | 3.79 | 66 | -22 | 32 |
| | Medial frontal gyrus (BA9) | 24 | 3.92 | 0 | 50 | 24 |
| | Inferior frontal gyrus (BA47) | 17 | 3.97 | -42 | 32 | -8 |

**Supplement figure S1**

The missed target contrast signal extracted from DLPFC divided on subgroups and zygosity. The numbers were too small to perform meaningful statistical analyses, but as illustrated, the dizygotic unaffected co-twins are the ones with the largest mean contrast signal whereas the mean contrast signal in the monozygotic co twins is more like the probands.

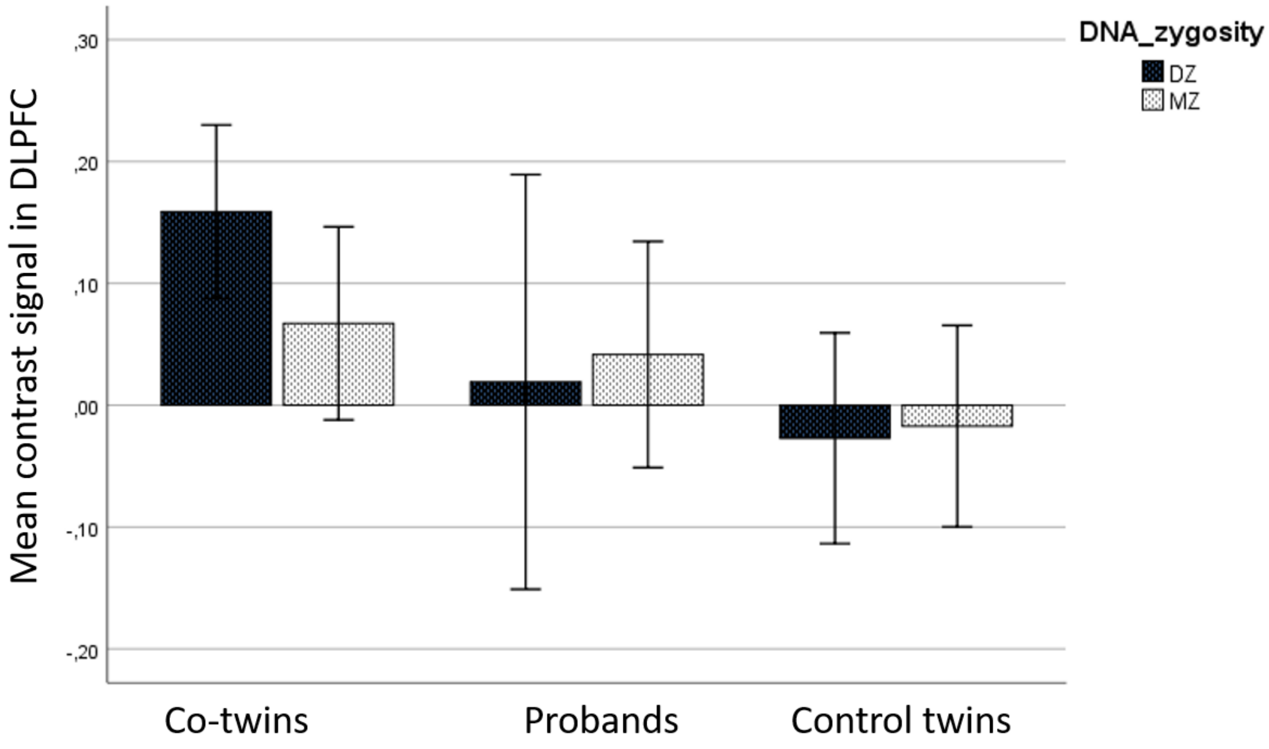

**Supplement table S3 Analyses on the schizophrenia only sample;** Clusters with group difference in whole brain analyses, thresholded Z>3.1

| Contrast | Group comparison | Area | Voxels | Z-max | X | Y | Z |
|---|---|---|---|---|---|---|---|
| Missed target | Co-twin > control | Occipital lope | 142 | 4.14 | 24 | -94 | -8 |
| | | Occipital lope | 133 | 4.35 | 28 | -86 | 12 |
| Monetary loss | Proband > control | Superior parietal lobe | 123 | 4.72 | 42 | -42 | 54 |
| Anticipation of winning | Control > proband | Frontal cortex | 194 | 4.78 | 42 | 42 | 36 |

**Supplement figure S2** Whole brain group comparison for the schizophrenia only sample during the missed target contrast where unaffected co-twins showed higher signal than control twins

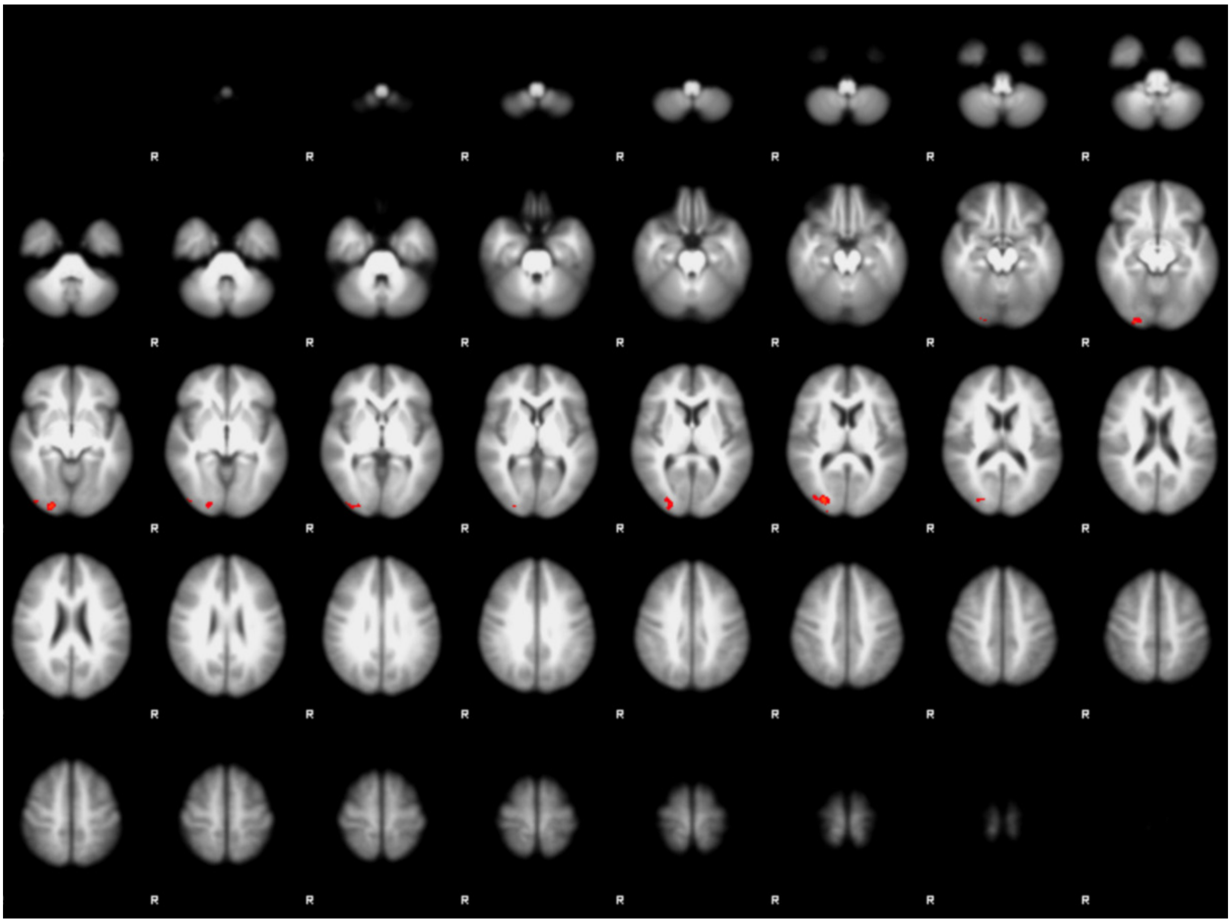

**Supplement figure S3** Whole brain group comparison for the schizophrenia only sample during the monetary loss contrast where probands showed higher signal than control twins

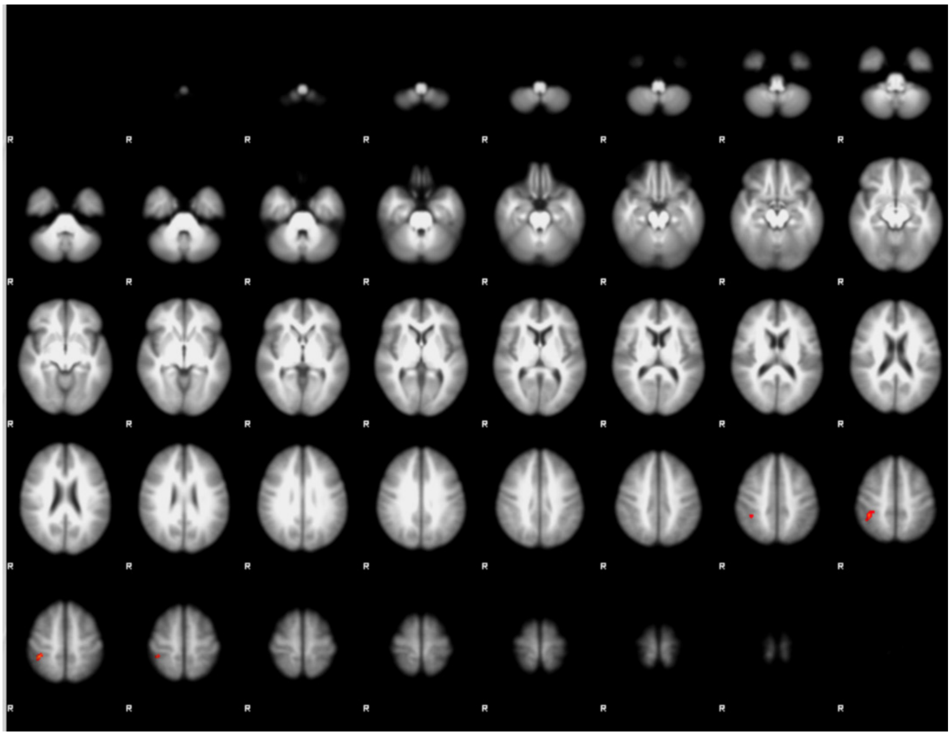

**Supplement figure S4** Whole brain group comparison for the schizophrenia only sample during the anticipation of winning contrast where control twins showed higher signal than probands

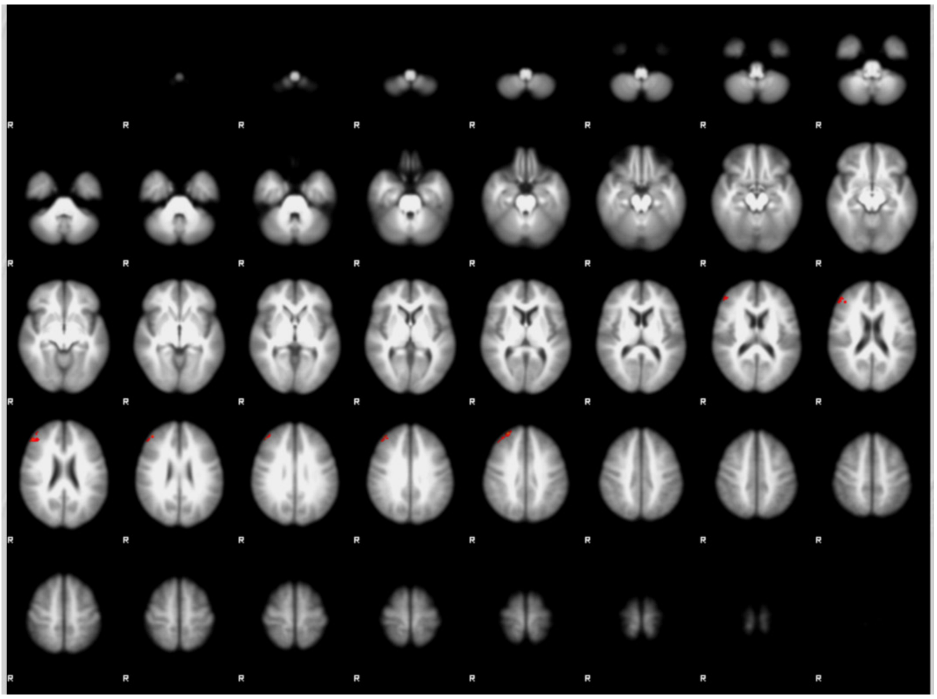

**Supplement figure S5** Extracted ROI values for the schizophrenia only sample. Error bars show 95% confidence interval.

A Signal from the anticipation of unpredictable outcome contras, left nucleus accumbens.

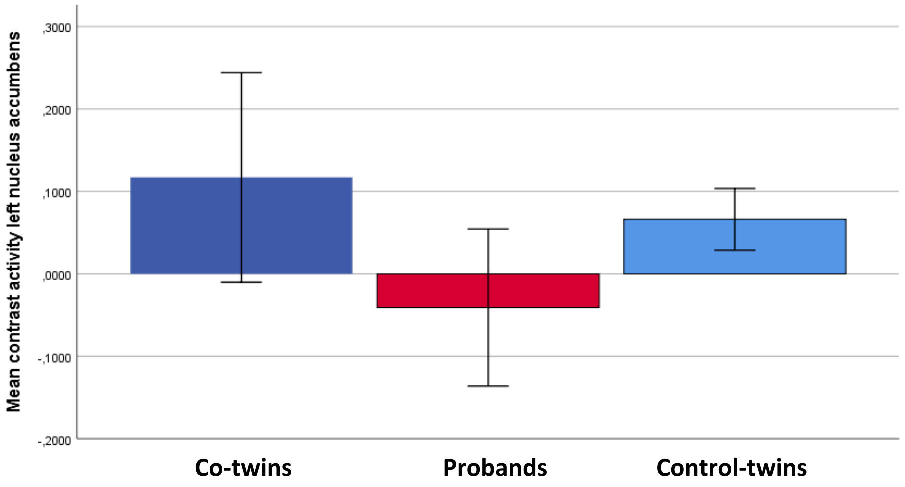


B Signal from the anticipation of unpredictable outcome contrast, right nucleus accumbens.

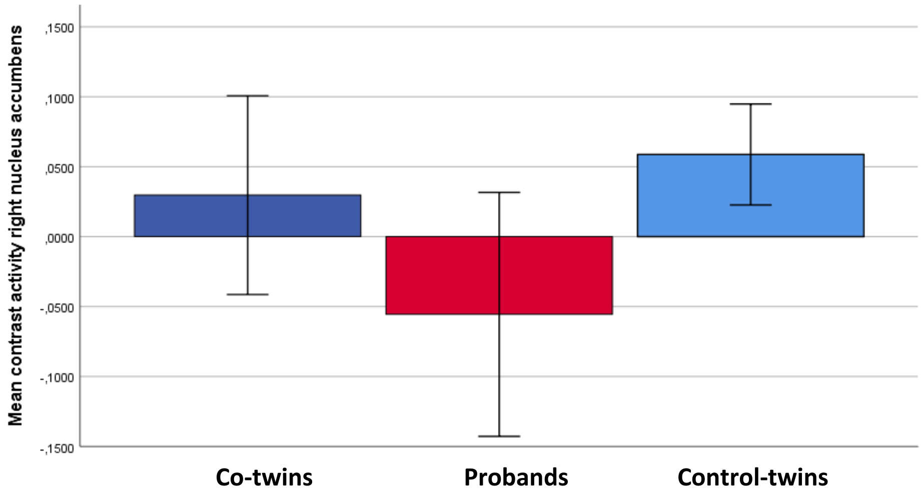


C Signal from the evaluation of the missed target contrast, right DLPFC

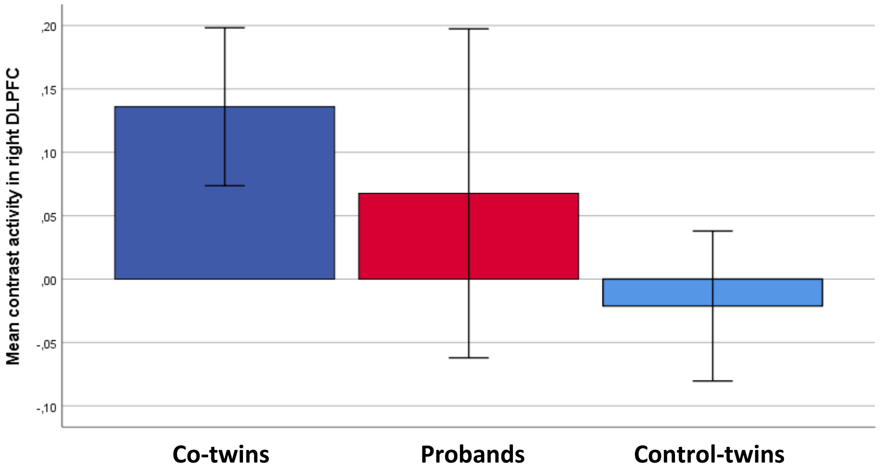

Supplement: Supplementary Tables 1 and 2, and Supplementary Figures 1–5 [file mmc1.pdf]
